# Ability of municipality-level deprivation indices to capture social inequalities in perinatal health in France: A nationwide study using preterm birth and small for gestational age to illustrate their relevance

**DOI:** 10.1186/s12889-022-13246-1

**Published:** 2022-05-09

**Authors:** Yaya Barry, Yann Le Strat, Elie Azria, Maud Gorza, Hugo Pilkington, Sthéphanie Vandentorren, Anne Gallay, Nolwenn Regnault

**Affiliations:** 1 Non-Communicable Diseases and Trauma Division, Santé Publique France, the French National Public Health Agency, 12, rue du Val d’Osne, 94415 Saint-Maurice, France; 2 Data Science Division, Santé Publique France, the French National Public Health Agency, Saint-Maurice, France; 3 Perinatal and Paediatric Epidemiology (EPOPé) Research Team, CRESS U1153, INSERM, Université de Paris, Paris, Obstetrical France; 4 Maternity Unit, Paris Saint Joseph Hospital, DHU Risks in Pregnancy, Paris Descartes University, Paris, France; 5 Health Promotion and Prevention Division, Santé Publique France, the French National Public Health Agency, Saint-Maurice, France; 6 Département de Géographie, Université Paris 8 Vincennes-Saint-Denis, UMR7533 Ladyss, Saint-Denis, France; 7 Scientific and International Division, Santé Publique France, the French National Public Health Agency, Saint-Maurice, France

**Keywords:** Deprivation indices, Social inequalities in health, Socioeconomic factors, Preterm birth, Small for gestational age

## Abstract

**Background:**

Evidence-based policy-making to reduce perinatal health inequalities requires an accurate measure of social disparities. We aimed to evaluate the relevance of two municipality-level deprivation indices (DIs), the French-Deprivation-Index (FDep) and the French-European-Deprivation-Index (FEDI) in perinatal health through two key perinatal outcomes: preterm birth (PTB) and small-for-gestational-age (SGA).

**Methods:**

We used two data sources: The French National Perinatal Surveys (NPS) and the French national health data system (SNDS). Using the former, we compared the gradients of the associations between individual socioeconomic characteristics (educational level and income) and “PTB and SGA” and associations between municipality-level DIs (Q1:least deprived; Q5:most deprived) and “PTB and SGA”. Using the SNDS, we then studied the association between each component of the two DIs (census data, 2015) and “PTB and SGA”. Adjusted odds ratios (aOR) were estimated using multilevel logistic regression with random intercept at the municipality level.

**Results:**

In the NPS (*N* = 26,238), PTB and SGA were associated with two individual socioeconomic characteristics: maternal educational level (≤ lower secondary school vs. ≥ Bachelor’s degree or equivalent, PTB: aOR = 1.43 [1.22–1.68], SGA: (1.31 [1.61–1.49]) and household income (< 1000 € vs. ≥ 3000 €, PTB: 1.55 [1.25–1.92], SGA: 1.69 [1.45–1.98]). For both FDep and FEDI, PTB and SGA were more frequent in deprived municipalities (Q5: 7.8% vs. Q1: 6.3% and 9.0% vs. 5.9% for PTB, respectively, and 12.0% vs. 10.3% and 11.9% vs. 10.2% for SGA, respectively). However, after adjustment, neither FDep nor FEDI showed a significant gradient with PTB or SGA. In the SNDS (*N* = 726,497), no FDep component, and only three FEDI components were significantly associated (specifically, the % of the population with ≤ lower secondary level of education with both outcomes (PTB: 1.5 [1.15–1.96]); SGA: 1.25 [1.03–1.51]), the % of overcrowded (i.e., > 1 person per room) houses (1.63 [1.15–2.32]) with PTB only, and unskilled farm workers with SGA only (1.52 [1.29–1.79]).

**Conclusion:**

Some components of FDep and FEDI were less relevant than others for capturing ecological inequalities in PTB and SGA. Results varied for each DI and perinatal outcome studied. These findings highlight the importance of testing DI relevance prior to examining perinatal health inequalities, and suggest the need to develop DIs that are suitable for pregnant women.

**Supplementary Information:**

The online version contains supplementary material available at 10.1186/s12889-022-13246-1.

## Background

Social deprivation can be measured at the ecological and individual levels [1–7]. For the former, publicly available aggregated variables are frequently used to measure socioeconomic status of inhabitants in residential areas [8, 9]. These variables are used in several ecological composite indices known as area-based deprivation indices [10–15] most of which are developed from census data. Previous studies showed that the way DIs are constructed may impact their ability to measure health inequalities (i.e., the data sources and socioeconomic variables selected, as well as the methods chosen to combine these variables into a single measure) [16–21]. The best possible data source meets all the following criteria: it reflects the deprivation experienced by the largest number of people in the most accurate and reliable way, it is the most recent source, it is reproducible, and its data are collected in a consistent manner throughout a country [21]. The most suitable geographic area is that with the smallest possible population size [20], while the most appropriate socioeconomic variables to explain social inequalities in health are those which are selected based on the theoretical model of the occurrence of the health problem studied [18]. Not all individual or neighbourhood-level socioeconomic variables are equally effective at uncovering health inequalities in different population groups [22].

In France, two DIs based on different methods and objectives are often used to measure social deprivation [13, 14]. The first is the French Deprivation Index (FDep) [14], which is built from four ecological variables to capture spatial variability of mortality. The second is the French European Deprivation Index (FEDI), which is used as a proxy of individual experience of deprivation, and is built from a combination of ten weighted census-derived elements [13].

For several years, these two municipality-level DIs have been used to measure social inequalities in perinatal health, particularly in preterm birth (PTB) and small-for-gestational-age (SGA) [23]. However, a previous study showed that some ecological variables (i.e., the socioeconomic variables of the municipality of residence) of the FDep appeared to be less relevant for perinatal health [4].

Among the different perinatal outcomes, PTB represents a large burden for families, healthcare, and educational systems in France [24, 25]. At 5½ years, 28% of children born extremely preterm have severe/moderate neurodevelopmental disabilities. This risk is more than three times higher in families with low socioeconomic status [25]. In addition, preterm infants are at risk of long-term neurocognitive, motor impairments and more risk of chronic diseases [26]. SGA is associated with a four-fold increased risk of stillbirth [27], and severe perinatal morbidity and mortality [28]. SGA is also associated to the ‘fetal origins of adult disease’ hypothesis with a higher risk of chronic diseases in later adulthood, such as cardiovascular diseases, metabolic syndrome, type 2 diabetes mellitus [29, 30]. The aetiologies of both outcomes are complex, and linked to a wide range of factors. Although the factors associated with these outcomes have been widely studied, mechanisms or pathways leading to PTB and SGA are still not fully known [3, 23, 31–45]. Risk factors often co-occur and their relationships with “PTB and SGA” may not be simple or direct [37]. Accordingly, adverse behaviours, such as tobacco smoking, and potentially protective factors, such as increased health literacy, may mediate the association between “PTB and SGA” and socioeconomic characteristics. Risk factors associated with “PTB and SGA” include sociodemographic and socioeconomic characteristics, individual chronic conditions and previous pregnancy-related medical risks (e.g., hypertension, infections, etc.), obstetric factors (e.g., parity, previous pregnancy outcomes, multiple births), foetal factors, psychosocial factors (e.g., social support, sense of community), environmental factors, mothers’ behaviours during pregnancy (e.g., illicit substance use and nutrition), and prenatal care [31, 34, 37–40, 44, 45]. Previous studies showed that individual socioeconomic characteristics such as educational level [3], household income [32, 36, 39], being a single-parent [43], working conditions [41], and demographic factors such as race and ethnicity [35, 42] were all associated with PTB and SGA, as well as contextual factors such as neighbourhood deprivation status [23].

Evidence-based policy-making for reducing social perinatal health inequalities requires measuring social disparities accurately and therefore validating DIs across subpopulations is important [17]. However, this is rarely done [4, 46, 47]. Furthermore, researchers do not often discuss or justify their decision to choose one area-based DI over another to measure health inequalities with respect to the outcomes or the characteristics of the subgroups studied.

In France, large medico-administrative databases are increasingly used in epidemiology for health surveillance. However, they lack information on individual socioeconomic status but include the municipality of residence, which allows assigning area-based DI values. DIs are then used as a proxy of individual socioeconomic status.

We aimed to investigate the ability of FDep and FEDI to measure social inequalities in perinatal health by i) comparing health inequalities captured by these two municipality-level DIs with those captured by individual socioeconomic characteristics in terms of two adverse perinatal outcomes, PTB and SGA, and ii) studying the association between each component of the two DIs and PTB and SGA.

## Methods

### Study design

We conducted a cross-sectional study in metropolitan France using two data sources: the French National Perinatal Surveys (NPS)—specifically the two most recent surveys in 2010 and 2016—and the French national health data system (SNDS). We used data from the NPS, which included all births that occur in a given week in France, as these data are representative of all births in France. The NPS also provide detailed data on mothers’ individual socioeconomic characteristics. The SNDS is an exhaustive national medico-administrative database. We used data from it to generalise our findings to all births in France.

### Data source

#### The French national perinatal surveys (NPS)

The NPS provide a population-based representative sample of births in France. They include all births in France occurring in a week, that is to say, all children born alive or still born in all public and private maternity units with a gestational age of at least 22 weeks, or weighing at least 500 g at birth [48]. In the present study, we used data for metropolitan France from the two most recent NPS surveys, specifically the NPS-2010 and NPS-2016. In the NPS, data on mothers’ demographic characteristics (e.g., maternal age), socioeconomic status (e.g., educational level, household income), prenatal care and behaviours (e.g., tobacco, cannabis or alcohol use), were collected during interviews in the postpartum ward, while data on deliveries and newborns’ health were extracted from medical records. The latter data included information on the mode of onset of delivery and any pre-existing maternal medical conditions. The National Council on Statistical Information (Comité du Label) and the French Commission on Information Technology and Liberties (CNIL) approved both NPS, assigning them the following numbers 2010X716SA (Comité du Label), 909,003 (CNIL) in 2010 and 2016X703SA (Comité du Label), 915,197 (CNIL) in 2016.

#### The French national health data system (SNDS)

Among other data, the SNDS contains data from the French health insurance database (*Données de consommation inter-régimes*, DCIR) and data from the French hospitalization activity database (*Programme de médicalisation des systèmes d’information*, PMSI-MCO) [49].

The DCIR contains a comprehensive and anonymous record of all outpatient visits, prescriptions, and reimbursements for out-of-pocket healthcare spending for more than 99% of population living in France. Reimbursable drugs are coded according to the Anatomical Therapeutic Chemical (ATC) classification. Medical and surgical procedures, prescribed medical devices, and biological examinations are also recorded, as is the FDep index.

The PMSI-MCO database records all inpatient data from public and private hospital admissions in medicine, surgery and obstetrics departments, including admission and discharge dates, principal diagnosis, related diagnosis and significant associated diagnoses. Diagnoses are coded according to the International Classification of diseases 10^th^ edition ICD-10 [50]. Gender, birthweight and gestational age are recorded in neonates’ hospital birth records.

#### Ecological deprivation indices

Data for all the various components of FDep and FEDI were taken from the 2015 French national census conducted by the National Institute of Statistics and Economic Studies (INSEE). Data for the median household income came from the national tax authority (www.insee.fr). The geographical unit used was the municipality*.* The FDep is constructed using principal component analysis based on four components (median household income, proportion of secondary school graduates among inhabitants aged 15 years and over, percentage of blue-collar workers in the active population, and proportion of unemployment). The FEDI is based on ten components from census data (proportions of non-home owners, unemployment, foreign nationals, persons with no access to a car, unskilled workers, households with ≥ 6 persons, primary residences with more than 1 person per room, persons with a low level of education, single-parent households, homes without exclusive use of indoor toilet/bath or shower) associated with an individual deprivation indicator. This individual deprivation indicator is defined based on the French version of the European Union Statistics on Income and Living Conditions (EU-SILC) study.

We used scores for both DIs calculated at the municipality scale from two editions of FDep (2009 and 2016) and two editions of FEDI (2011 and 2015). Only data from metropolitan France were used, as FDep scores are not available for French overseas territories.

### Study population

#### NPS study population

**Fig. 1:**
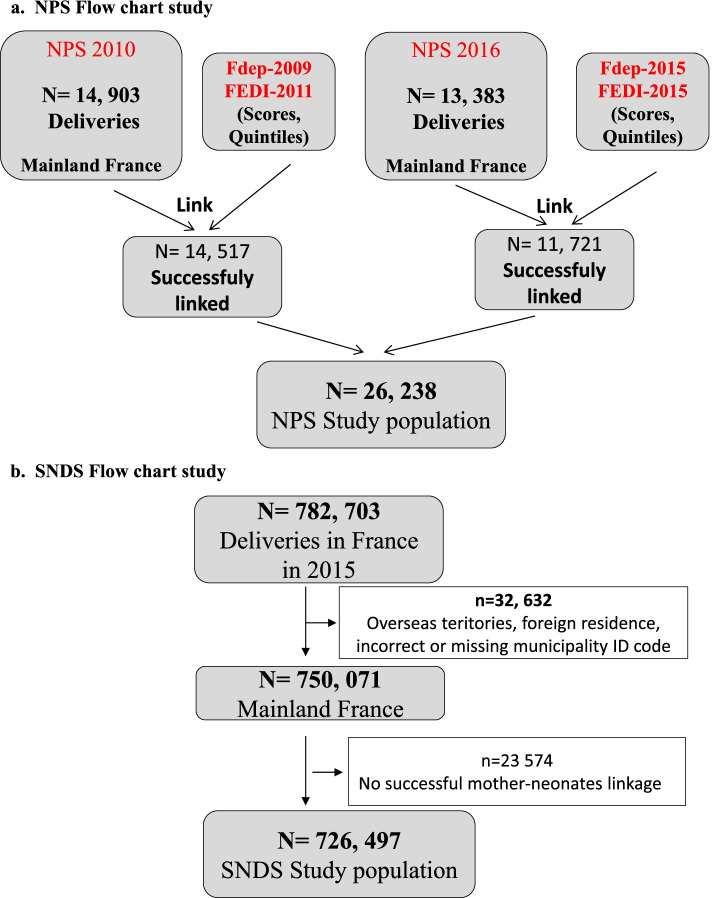
Flow chart of the study

In order to constitute the sample, we assigned a score and quintile of each DI to each mother in each of the two NPS databases using the identifier number of the municipality of residence if available, or alternatively, using the municipality name (Fig. 1). To take into account possible temporal changes in municipalities’ socioeconomic characteristics, we assigned the NPS data to the closest editions of DIs. Accordingly, NPS-2010 was assigned to the FDep 2009 and FEDI 2011 scores, and NPS-2016 to the FDep 2016 and FEDI 2015 scores. A total of 26, 238 births recorded in the NPS were included in the present study out of a total of 1,533,412 births recorded in metropolitan France for 2010 and 2016.

#### SNDS study population

We included all deliveries after 22 gestational weeks in metropolitan France in 2015 identified in the SNDS database successfully linked with socioeconomic characteristics of mothers’ municipality of residence (census data, 2015) and with 2015 household incomes as declared to the tax authority (Fig. 1). Deliveries were identified using hospital diagnoses (Z37) coded according to the ICD-10 [50]. We excluded all deliveries without successful linkage between maternal and neonatal data from the analysis (3.1%). The SNDS population included in the present study comprised 726,497 births out of the 754, 756 births recorded births in metropolitan France in 2015.

### Outcomes

Studied outcomes were PTB and SGA. PTB was defined as birth occurring prior to a gestational age of 37 completed weeks. In the NPS analysis, we used the gestational age recorded in medical files on deliveries, while in the SNDS analysis we used the gestational age recorded in mothers’ maternity ward hospitalisation records when available, or alternatively, from neonates’ hospital birth records. SGA births were defined according to French foetal growth curves references [51] which provide expected gender-specific distributions of birth weight according to gestational age. Neonates were deemed SGA if their weight was below the 10^th^ percentile of this national reference.

### Covariables

In the NPS analysis, individual-level socioeconomic status was assessed using mothers’ educational level, household income and the individual social deprivation index (ISDS). More specifically, educational level was defined based on the International Standard Classification of Education (ISCED) 2011 [52]: low educational level-ISCED 0–2 (i.e., up to lower secondary education), medium educational level-ISCED 3 (i.e., upper secondary education), post-secondary educational level-ISCED 4–5 (i.e., post-secondary non-tertiary, short cycle tertiary), high educational level-ISCED 6–8 (university Bachelors’ degree equivalent or higher). Household monthly income was classified into four categories (< 1000€; [1000€—2000€ [; [2000€ -3000€ [; ≥ 3000€). The individual social deprivation index [6] was calculated as the sum of the following four socioeconomic characteristics: receiving the active solidarity income government allowance (RSA, *Revenu de Solidarité Active*); benefitting from the universal health care cover (CMU, *Couverture maladie universelle*, which is provided to all those legally continuously residing in France for more than three months) *or* not having social health insurance; not living in one’s own accommodation; and not living with a partner [6]. The value 0 was given to each characteristic when absent or 1 when present. We used continuous and categorical (i.e., quintile) scores: Q1 and Q5 corresponded to the least and most deprived areas, respectively. Other variables were maternal age, parity, previous PTB, previous SGA, other adverse obstetric history (previous stillbirths and neonatal mortality), mother’s birth country, tobacco use, cannabis use, and pre-pregnancy body mass index (BMI) (kg/m^2^) in four classes (< 18.5; 18.5–24.9; 25–29.9; ≥ 30).

In the SNDS analysis, covariables were identified using algorithms based on hospital ICD-10 diagnoses and/or drug reimbursements coded according to the Anatomical Therapeutic Chemical (ATC). These algorithms are described in Appendix 1. Individual-level socioeconomic position was assessed by whether or not the mother was receiving French universal complementary medical coverage (CMU-C, *Couverture maladie universelle complémentaire,* which is provided to persons with a monthly income limit of < 721€), maternal deprivation (defined as meeting at least one of the following three socioeconomic characteristics coded according to the International Classification of Diseases 10^th^ edition: problems related to education and literacy; problems related to economic circumstances, and problems related to inadequate housing). Other variables were maternal age, parity, previous PTB, previous SGA, previous tobacco consumption, obesity, and previous hypertension (Appendix 1).

### Statistical analyses

Categorical variables were expressed as percentages and continuous variables as medians with interquartile ranges. We quantified the degree to which FDep and FEDI classified municipalities into the same or a close quintile using weighted Cohen’s Kappa (Kw) statistic.

In the NPS-2010 and NPS-2016 databases, we studied associations between the DIs’ quintiles and PTB and SGA. Multilevel logistic regression models were used to estimate adjusted odds ratios (aOR) and their 95% confidence intervals with random intercept at the municipality level. We built six models (three for FDep and three for FEDI) for each outcome (PTB and SGA) by separately including mother’s educational level, monthly household income, and mother’s individual social deprivation index (Appendix 2).

In the SNDS analysis, we studied the associations between both DIs’ components and PTB and SGA, using multilevel logistic regression models. We used one model for each component of the two DIs. The dependent variables were PTB and SGA and the covariables were each of the components of FDep and FEDI and mothers’ individual characteristics. Finally, all significant components of FDep and FEDI were introduced into one multilevel model (Appendix 2).

Covariables included in all models were selected a priori from the literature [3, 23, 33, 37–40, 44] or based on Directed Acyclic Graphs (DAGs) [31]. We did not include pregnancy complications (including preeclampsia/eclampsia) with a strong deterministic association with PTB/SGA in our analyses, as they constitute intermediate variables between mother’s socioeconomic characteristics and the risk of PTB and SGA [31, 37, 38]. Despite the fact that tobacco smoking appears to be a mediator of socioeconomic differences in “PTB and SGA” [53], we decided to include it in the mains analyses to evaluate the association between “PTB and SGA” and direct risk factors independent of health behaviours during pregnancy. We did not introduce alcohol consumption into the statistical analyses. Even though the information was collected, it was considered as underestimated compared to the actual consumption in the NPS-2016 according to the France Health Barometer – 2017 [54]. All tests for statistical significance used a two-sided α error of 0.05. NPS analyses were performed using Stata 14.2® software, (College Station, Texas 77,845 USA) and SNDS analyses using SAS Entreprise Guide software, version 7.1® (SAS Institute Inc., Cary, NC, USA).

## Results

### Description of the study population, individual characteristics, individual socioeconomic status and DIs according to PTB and SGA

The final NPS (i.e., the 2010 and 2016 NPS combined) study population included 26,238 women. Approximately 2% were less than 20 years old, while 20.2% were 35 years old or over. Three-quarters had at least completed upper secondary school (ISCED 3) (74.3%), 10% had a household monthly income of less than 1000 €, and 4% had an individual social deprivation index greater than or equal to three. The overall rates of PTB and SGA were 7.1% and 11.1%, respectively (Table 1).

**Table 1:** Individual characteristics and deprivation indices, overall and by outcome (preterm birth (PTB), small for gestational age (SGA)), Metropolitan France, National Perinatal survey, 2010–1016

	**Total**		**PTB**			**SGA**		
	**%**	**n**	**%**	**n**	***p***^***a***^	**%**	**n**	***p***^***a***^
N		26,238	7.1	1845		11.1	2908	
**Maternal age (years)**					**0.004**			** < 0.001**
< 20	1.9	508	11.0	55		15.9	79	
[20–25[	13.1	3421	7.4	253		13.3	453	
[25–30[	32.4	8498	6.9	586		10.9	920	
[30–35[	32.4	8491	6.7	566		10.2	861	
≥ 35	20.2	5296	7.3	384		11.3	595	
**Educational level**					** < 0.001**			** < 0.001**
Lower secondary education (ISCED 0–2)	25.7	6613	8.3	545		13.6	893	
Upper secondary education (ISCED 3)	20.5	5270	7.1	371		10.9	573	
post-secondary non-tertiary, short cycle tertiary (ISCED 4–5)	20.6	5300	6.7	356		10.3	545	
university Bachelors’ degree equivalent or higher (ISCED 6–8)	33.2	8532	5.8	490		9.6	816	
**Household income (€)**					** < 0.001**			** < 0.001**
< 1000	9.7	2442	8.9	215		15.5	375	
[1000–2000[	23.1	5786	7.3	421		12.0	688	
[2000–3000[	29.2	7317	6.8	498		10.8	790	
≥ 3000	38.0	9531	5.5	521		9.4	890	
**Individual deprivation index**					** < 0.001**			** < 0.001**
0	78.8	20,322	6.4	1299		10.3	2078	
1	10.6	2725	8.1	221		12.7	345	
2	6.6	1691	8.3	139		13.7	229	
≥ 3	4.1	1064	8.8	93		16.4	173	
**Previous PTB**					** < 0.001**			** < 0.001**
No	96.3	24,522	6.4	1574		10.9	2653	
Yes	3.7	938	23.4	219		18.2	170	
**Previous SGA**					** < 0.001**			** < 0.001**
No	96.6	24,569	6.5	1660		10.5	2580	
Yes	3.4	872	15.2	132		27.6	240	
**Parity**					** < 0.001**			** < 0.001**
No	57.1	14,917	6.5	962		8.9	1327	
Yes	42.9	11,232	7.8	875		14.0	1567	
**BMI before pregnancy (kg/m**^**2**^**)**					** < 0.001**			** < 0.001**
< 18.5	7.8	1983	8.7	173		17.1	338	
18.5–24	62.9	15,873	6.3	999		11.1	1749	
25–29.9	18.5	4677	1.2	304		9.4	438	
≥ 30	10.8	2718	7.7	208		9.0	243	
**Maternal birth country**					**0.0004**			**0.01**
France	81.7	21,075	6.6	1377		11.2	2351	
Other European	3.9	1001	6.4	64		9.6	95	
North Africa	7.0	1814	7.5	136		9.0	162	
Sub-Saharan Africa	4.4	1147	9.4	107		12.3	141	
Other	3.0	765	8.8	67		10.3	78	
**Smoking** (number of cigarettes/day during the third trimester)					**0.02**			** < 0.0001**
0	83.5	21,543	6.6	1428		9.3	2002	
1–9	12.1	3118	7.4	231		19.1	592	
≥ 10	4.4	1133	8.4	95		21.6	243	
**Cannabis consumption**								** < 0.0001**
No	98.4	24,907	6.5	1639		10.8	2679	
Yes	1.6	392	9.5	37		21.5	83	
**Other adverse obstetric history**					** < 0.0001**			** < 0.0001**
No	92.2	23,547	6.3	1476		10.4	2445	
Yes	7.8	1979	16.4	324		19.2	379	
**Urban–Rural status of munipalities**					**0.001**			**0.04**
Isolated rural with very low density	3.1	744	6.3	47		10.5	78	
Isolated rural with low density	10.4	2536	6.7	169		12.8	324	
Rural with low relationship with an employment hub	9.2	2244	5.6	125		10.2	227	
Rural with strong relationship with an employment hub	10.4	2535	6.8	172		10.3	261	
Suburban (intermediate density)	26.8	6520	6.9	446		11.2	727	
Urban centre (highly urban)	40.0	9712	7.9	767		11.3	1087	
**FDep (quintile)**					**0.03**			**0.02**
Q1	20.06	5264	6.3	329		10.3	541	
Q2	19.73	5176	6.8	349		11.0	566	
Q3	20.02	5254	7.2	378		10.6	553	
Q4	19,73	5178	7.2	370		11.8	606	
Q5	20,45	5366	7.8	419		12.0	642	
**FEDI (quintile)**					** < 0.0001**			**0.03**
Q1	19.99	5245	6.0	312		10.2	530	
Q2	19.94	5231	6.5	343		10.8	560	
Q3	20.02	5252	7.0	370		11.1	582	
Q4	20.04	5259	6.7	353		11.8	617	
Q5	20.01	5251	9.1	474		11.9	619	

^a^Chi2 test

PTB and SGA rates were higher for mothers with a low educational level (ISCED 0–2), low-income households, and higher individual social deprivation index (Table 1). Both outcomes were more frequent in more socially deprived municipalities when using the FDep index, (Q5: 7.8% vs. Q1: 6.3% and 12.0% vs. 10.3% respectively) and the FEDI (PTB 9.1% vs. 6.0%; SGA: 11.9% vs. 10.2%).

The final SNDS study population included 726,497 deliveries. The overall PTB and SGA rates were 7.5% and 11.9%, respectively. The population characteristics are presented in Table 2.

**Table 2:** Maternal characteristics, overall and by outcome (preterm birth (PTB), small for gestational age (SGA)), Metropolitan France, SNDS, 2015

	**Total**		**PTB**			**SGA**		
	**%**	**n**	**%**	**n**	***p***^***a***^	**%**	**n**	***p***^***a***^
N	726,497		7.5	54,378		11.9	86,305	
**Maternal age (years)**					**0.004**			** < 0.001**
< 20	1.9	13,950	9.2	1,285		16,2	2,256	
[20–25[	12.4	89,934	7.7	6,877		13,7	12,290	
[25–30[	31.7	230,368	7.1	16,288		11,6	26,676	
[30–35[	33.4	242,236	7.0	17,032		11,2	27,208	
≥ 35	20.7	150,009	8.6	12,896		11,9	17,875	
**CMU-C**					** < 0.001**			** < 0.001**
No	81.0	588,406	7.2	42,207		11,5	67,769	
Yes	19.0	138,091	8.8	12,171		13.4	18,536	
**Previous PTB**					** < 0.001**			**0.002**
No	97.0	704,345	7.1	50,092		11.8	83,529	
Yes	3.0	22,152	19.4	4,286		12.5	2,776	
**Previous SGA**					** < 0.001**			** < 0.001**
No	98.3	714,491	7.4	53,135		11.6	83,122	
Yes	1.7	12,006	10.4	1,243		26.5	3,183	
**Parity**					** < 0.001**			** < 0.001**
No	44.4	322,349	8.7	28,023		6.8	49,295	
Yes	55.6	404,148	6.5	26,355		9.2	37,010	
**obesity** **(≥ 30)**					** < 0.001**			** < 0.001**
No	95.0	689,894	7.3	50,646		11.4	82,509	
Yes	5.0	36,603	10.2	3,732		10.4	3,796	
**Previous tobacco consumption**					** < 0.001**			** < 0.0001**
No	90.5	657,501	7.2	47,460		10.0	72,594	
Yes	9.5	68,781	10.0	6,903		19.9	13,679	
**Previous hypertension**					** < 0.0001**			** < 0.001**
No	98.4	714,984	7.3	52,441		11.7	83,947	
Yes	1.6	11,513	16.8	1,937		20.5	2,358	
**Individual maternal deprivation**					** < 0.0001**			** < 0.0001**
No	98.1	713,062	7.4	52,924		11.8	84,235	
Yes	1.9	13,435	10.8	1,454		15.4	20.7	

^a^Chi2 test

### Agreement between DIs

We observed moderate agreement (Kw = 0.43) between FDep and FEDI in terms of the classification of municipalities in the various quintiles (Appendix 3).

The FEDI classified large majority of the ‘highly urban municipalities’ in the more deprived quintile (62.3%) compared with 15,5% for the FDep (Appendix 3). Regarding the ‘isolated rural municipalities with very low density’, 22.3% were classified in the most deprived quintile for FEDI and 27.7% for FDEP (Appendix 3).

### DIs’ ability to capture perinatal health inequalities

**Fig. 2:**
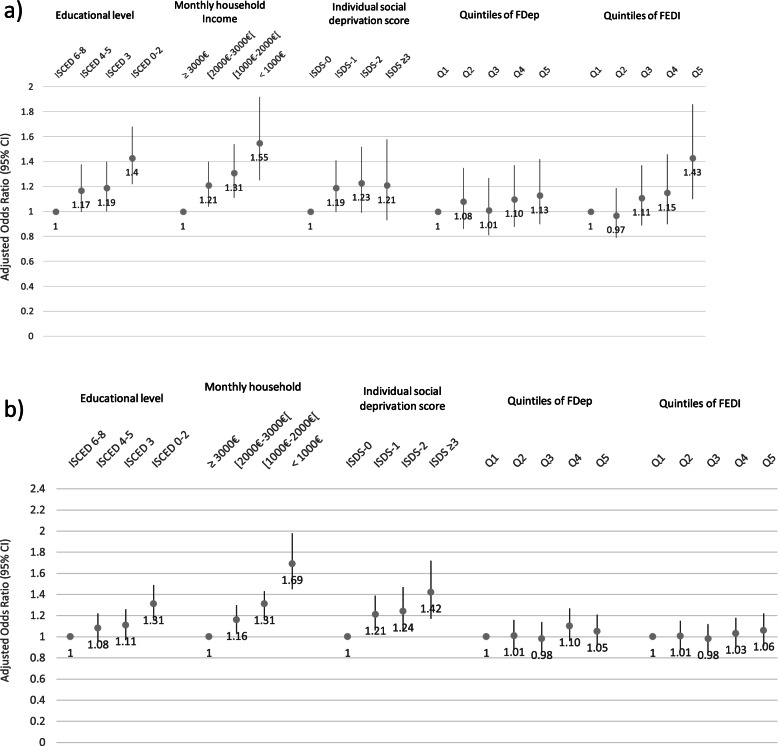
Multivariate multilevel analyses for the NPS: Association between PTB and individual socioeconomic status, and the quintiles of both DIs (FDep, FEDI) (Fig. 2a). Association between SGA and individual socioeconomic status, and the quintiles of the DIs (FDep, FEDI) (Fig. 2 b). aOR (95% CI) = adjusted odds ratio (95% confidence interval) from multilevel logistic regression models. Educational level categorized into four classes: ISCED 0–2 (lower secondary education or less), ISCED 3 (upper secondary education), ISCED 4–5 (post-secondary non-tertiary education, short-cycle tertiary education), ISCED 6–8 (university Bachelors’ degree or equivalent or higher). Individual social deprivation index categorized into in four classes: ISDI-0: zero factor, ISDI-1: one factor, ISDI-2: two factors, ISDI ≥ 3 factors. FDep/FEDI: Q1: least-deprived quintile (reference); Q5: most-deprived quintile. **a** Preterm Birth. **b** Small for gestational age

In the NPS analyses, we found a consistent inverse relationship between PTB, SGA and both mother’s educational level and household income with a significant gradient (Fig. 2a, b). The lower the educational level or monthly household income, the higher the risk of PTB and SGA. We also found a significant gradient between the individual social deprivation index and SGA, but no significant gradient for PTB.

When studying the DIs, after adjustment, no significant gradient was found between FDep or FEDI quintiles and either PTB or SGA (Fig. 2 a and b).

In the SNDS study, in individual univariate analysis, FDep and FEDI scores were associated with both PTB (OR = 1.03 [1.02–1.03] and 1.00 [1.00–1.01], respectively) and SGA (1.03 [1.03–1.04] and 1.002 [1.000–1.003], respectively), despite estimates being close to 1. In multivariate multilevel analyses, only three components of FDep (proportion of unemployment, median household income and percentage of blue-collar workers) were significantly associated with PTB and SGA (Appendix 4). With regard to FEDI components, neither the proportion of foreign nationals nor the proportion of households with no access to a car was associated with either PTB or SGA. The proportion of residences with more than one person per room was only associated with PTB (Appendix 5).

**Fig. 3:**
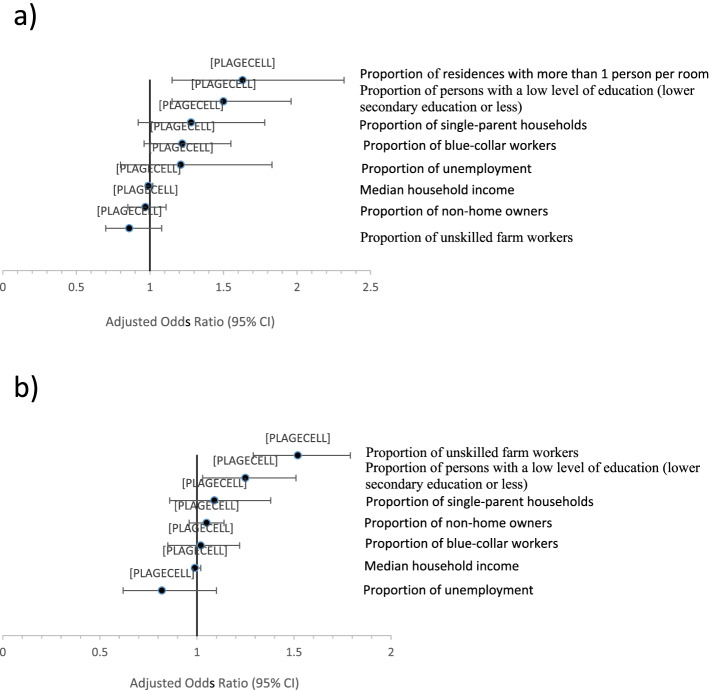
Multivariate multilevel analysis for both FDep and FEDI components in the SNDS: Association between FDep and FEDI components and preterm birth (Fig. 3a) and small for gestational age (Fig. 3b) which were significant in Appendices 4 and 5. aOR (95% CI) = adjusted odds ratio (95% confidence interval) from multilevel analyses. All FDep and FEDI significant components adjusted for individual mother’s characteristics in the same model. **a** Preterm birth. **b** Small for gestational age

When we used all significant DI components in the same multilevel model, no component of FDep was significantly associated with PTB or SGA. With regard to the FEDI, only three components remained statistically significant (Fig. 3). Specifically, the proportion of persons with lower secondary level of education or less was associated with both PTB and SGA, while the proportion of residences with more than one person per room was associated with PTB only, and the proportion of unskilled farm workers with SGA only (Fig. 3).

Neither the results for the proportion of households without exclusive use of indoor toilet/bath or shower nor those for the proportion of overcrowded households (i.e., ≥ 6 persons) are presented as these events were extremely rare.

## Discussion

Our findings showed that educational level and household income were associated with PTB and SGA. At the municipality level, no significant social gradient was observed between FDep and FEDI quintiles for either PTB or SGA. Only three of the DIs’ components were significantly associated with the two outcomes (proportion of persons with lower secondary education level or less, proportion of residences with more than 1 person per room for PTB, proportion of persons with lower secondary education level or less, proportion of unskilled farm workers for SGA, with one common component).

### Moderate agreement between FDep and FEDI

The FEDI tended to classify urban municipalities in more deprived quintiles, whereas the FDep tended to classify them in less deprived quintiles. This difference could be partly explained by the fact that the FDep and FEDI have different objectives and were built using different statistical methods. The FEDI is built from a combination of ten weighted census-derived elements as a proxy of individual experience of deprivation [13]. In contrast, the FDep is built from four ecological variables to capture spatial variability using the statistical procedure of principal component analysis and validated on mortality [14]. In addition, the different components in the two DIs may explain this difference. Previous studies showed that some components of the FEDI—such as the proportion of non-home owners, the proportion of primary residences with more than 1 person per room, and the proportion of persons with no access to a car—vary between rural and urban areas [55], and do not always reflect the deprivation status in an area of residence. In rural areas, not having access to a car could be an obstacle to mobility and may reflect deprivation status, whereas in urban areas, it is common not to have a car as public transport is developed. Likewise, while the proportion of overcrowded homes and home ownership may be markers of deprivation in rural areas, this is not necessarily true in urban contexts. When DI quintiles were applied to the NPS population, 60% of women were classified into the most deprived area according to FEDI quintiles (Q5) (data not shown). This could be explained by the fact that pregnant women are more often young, actively working and mainly live in urban areas.

### Disability to capture socioeconomic inequalities in the risk of PTB and SGA

With regard to individual socioeconomic characteristics, the significant associations we observed between household income and educational level with both PTB and SGA are consistent with previous studies [3, 33, 39]. Our results showed a less clear link between the composite variable ‘individual social deprivation index’ and PTB, despite an excess risk as soon as the score was higher than 0. This result is similar to that observed by Opatowski M et al. who built the individual social deprivation index we used here [6]. One explanation for this may be the small number of premature infants with a score >  = 3 (*n* = 93 PTB neonates), which prevents any potential significant difference from being demonstrated. Another explanation is PTB’s multifactorial aetiology, especially psychological status, behaviours (tobacco, cannabis and alcohol consumption, etc.), inadequate prenatal care, and commuting, which are all very important during pregnancy, but are not included in the index [37]. In the SNDS analyses, with regard to PTB, the non-significant associations we found for the proportion of blue-collar workers, the median household income, and the proportion of secondary school graduates, confirm those reported in a previous French study [4]. However, unlike that study, we found that the proportion of unemployment, the proportion of non-home owners, and the proportion of single-parent households were not associated with PTB. This may be due to differences in the analyses methods used [4]. More specifically, unlike previous analyses [4], we used multilevel regressions adjusted for a large range of variables, which allowed to study both individual and ecological variables.

We did not observe a significant association between the median household income and either PTB or SGA. This could be due to the difficulties in accurately measuring income, and especially financial burden, including household expenses and the number of dependent persons [36]. In contrast to the proportion of secondary school graduates, the proportion of persons with a lower secondary school level of education or less was associated with PTB and SGA. The high percentage of the general population who complete upper secondary educational level and the continued increase in this percentage over recent years could explain this result. With regard to the proportion of foreign nationals, our study did not show any significant link with PTB or SGA. In the literature, poor perinatal outcomes are not always associated with foreign nationality because of the ‘healthy migrant effect’ on first generation immigrants [56]. The association between components of both DIs and PTB and SGA were not always similar, perhaps due to difference in risk factors regarding the two outcomes [38, 39].

During the 2020 COVID-19-related lockdowns, the rate of PTB decreased in some countries, including Ireland [57], the Netherlands [58], the United Kingdom [59], and Denmark [60]. Some authors suggest that this decrease could be the result of cumulative effects of socio-environmental variables (such as maternal behavioural modifications, reduced work-related stress, possible alleviation of commuting-related and other work-related physical strain thanks to working from home), a likely increase in partner presence and support at home, reduced exposure to infection because of physical distancing measures and self-isolation, and less air pollution. Lockdowns and other preventive measures taken by governments against the pandemic, provide the opportunity to evaluate these and other risk factors in detail, for example geographical distance from healthcare service providers (which may increase difficulties in accessibility for some women), inadequate health care provision, distance from home to work, modes of transportation between home and work, rural–urban status, arduous working conditions, and insufficient infrastructure near one’s home to practice physical activity. It is also possible to evaluate the relevancy of taking these factors into account when constructing area-based deprivation indices. Finally, preventive measures also provide the opportunity to examine causal mechanisms related to perinatal outcomes.

Area based deprivation indices are also developed in France for smaller geographical areas than municipalities [12, 61]. However, it appears important to continue building area-level deprivation indices using relevant data, in the finer scale, which groups inhabitants with homogeneous contextual characteristics.

### Strengths and limitations

Our study has several strengths. France’s NPS are population-based and provide detailed data on mothers’ medical, social and demographic characteristics in a representative annual sample of births in France. We combined data from two French NPS (2010 and 2016) to increase the sample size. Our second study population was based on an exhaustive national medico-administrative database (SNDS), which includes all deliveries in France. We used multilevel models with random intercept to take into account variations at the municipality scale, and to study both individual and ecological variables. Our results may be generalised in the French context because they were based on data from a representative sample and from exhaustive national databases. In the international context, the methods used in our study could be reproduced to evaluate the validity of local deprivation indices.

The study also has limitations. First, we studied characteristics of residence areas at the municipality scale, as this is the scale available in the SNDS. While the *Îlot regroupé pour l’information statistique* (IRIS) system would have been a more ideal choice, as it is a finer scale and groups inhabitants with more homogeneous characteristics, it is not available in the SNDS. Second, the NPS analyses were designed to evaluate the extent that ecological deprivation indices can be considered good proxies of individual socioeconomic characteristics. Although this approach has been previously used in several studies [13, 47, 62, 63], one cannot simply interpret these indices as exclusively proxies for individual characteristics; they may also reflect the effects of the area of residence. Third, due to a lack of available data in the SNDS, we were not able to include as many individual covariables on maternal socioeconomic status as in the NPS analysis. Therefore, we used a different set of covariables on the NPS and SNDS multivariate analyses. To our knowledge, no study to date has assessed the validity of coding of the few available proxies of maternal individual-level socioeconomic deprivation in the SNDS database, limiting its potential usefulness.

## Conclusions

Two often-used deprivation indices in France, FDep and FEDI did not appear to be relevant to capture inequalities in either preterm birth or small for gestational age. Results varied according to the index and the perinatal outcome, which highlights the importance of testing a deprivation index’s validity prior to examining perinatal health inequalities, and suggests the need for developing indices that are relevant for pregnant women.

## Supplementary Information

**Additional file 1: Appendix 1.** Algorithms of definition of covariables in the SNDS. Algorithms of the main exposure in the SNDS analyses.

**Additional file 2: Appendix 2.** SNDS and National perinatal surveys analyses: Multilevel models. Presentation of all covariables of the different multilevel models used in the NPS and SNDS statistical analyses.

**Additional file 3: Appendix 3.** Maps of the quintiles of the municipalities according to FDep and FEDI indices ; Distribution of municipalities’ urban–rural status according to the quintiles of FDep and FEDI. Classification of municipalities in the various quintiles of FDep and FEDI and Distribution of municipalities’ urban–rural status according to the quintiles of FDep and FEDI.

**Additional file 4: Appendix 4.** Multivariate multilevel analyses for the FDep components in the SNDS. Association between FDep components and preterm birth and small for gestational age.

**Additional file 5: Appendix 5.** Multivariate multilevel analyses for the FDep components in the SNDS. Association between FEDI components and preterm birth and small for gestational age.

**Additional file 6. **FDep and FEDI Components at municipality. Data on the proportions of each components of FDep and FEDI analysed in this study.

## Data Availability

Due to third party restrictions, NPS and SNDS data supporting the findings of this study are not publicly available. Please see the following URL for more information: https://www.enp.inserm.fr/;https://www.snds.gouv.fr/SNDS/Accueil. Data on the components of FDep and FEDI analysed in this study are included within the article (and its additional file(s)).

## Abbreviations

ATC: Anatomical Therapeutic Chemical classification
aOR: Adjusted odds ratios
BMI: Body mass index
CMU: Couverture maladie universelle
CMU-C: Couverture maladie universelle complémentaire
CNIL: Commission on Information Technology and Liberties
DCIR: Données de consommation inter-régimes
DIs: Area-based deprivation indices
EU-SILC: European Union Statistics on Income and Living Conditions
FDep: French Deprivation index
FEDI: French European Deprivation Index
INSEE: National Institute of Statistics and Economic Studies
IRIS: Îlot regroupé pour l’information statistique
ISCED: International Standard Classification of Education
ISDI: Individual Social Deprivation Index
NPS: French national perinatal surveys
PMSI-MCO: Programme de médicalisation des systèmes d’information
PTB: Preterm Birth
Q: Quintile
RSA: Revenu de Solidarité Active
SGA: Small for gestational age
SNDS: French national health data system
