# Supplementary material for: Ability of municipality-level deprivation indices to capture social inequalities in perinatal health in France: A nationwide study using preterm birth and small for gestational age to illustrate their relevance
Source: BMC Public Health. 2022 May 9;22:919. doi: 10.1186/s12889-022-13246-1 (PMC9082984; doi:10.1186/s12889-022-13246-1)
Supplement: Supplementary file 1 — Additional file 1: Appendix 1. Algorithms of definition of covariables in the SNDS. Algorithms of the main exposure in the SNDS analyses. [file 12889_2022_13246_MOESM1_ESM.pdf]

Appendix 1 : Algorithms of definition of covariables in the SNDS

Variables	Algorithms: ICD-10 codes, Drug reimbursements (coded according to the Anatomical Therapeutic Chemical (ATC)
**Individual maternal deprivation**	
Problems related to education and literacy	Z55.0: Illiteracy and low-level literacy<br>Z55.1: Schooling unavailable and unattainable<br>Z55.8: Other problems related to education and literacy, Inadequate teaching<br>Z55.9: Problem related to education and literacy, unspecified
Problems related to economic circumstances	Z59.5: Extreme poverty<br>Z59.6: Low income
Inadequate housing	Z59.0: Homelessness;<br>Z59.1: Inadequate housing<br>Z59.8: Other problems related to housing and economic circumstances<br>Z59.9: Problem related to housing and economic circumstances, unspecified,
**Parity**	**Parity was coded in the delivery hospital stay for women who delivered vaginally (2015)**<br>JQGD001: Single breech vaginal birth by a woman who has previously given birth<br>JQGD002: Multiple vaginal birth by a woman giving birth for the first time<br>JQGD003: Single breech vaginal birth with arms extracted before head by a woman giving birth for the first time<br>JQGD004: Single breech vaginal birth by a woman giving birth for the first time<br>JQGD005: Single breech vaginal birth with breech extraction by a woman who has previously given birth<br>JQGD007: Multiple vaginal birth by a woman who has previously given birth<br>JQGD008: Single breech vaginal birth with arms extracted before head by a woman who has previously given birth<br>JQGD010: Single cephalic vaginal birth by a woman giving birth for the first time<br>JQGD012: Single cephalic vaginal birth by a woman who has previously given birth<br>JQGD013 : Single breech vaginal birth with breech extraction by a woman giving birth for the first time<br>**For women who had a cesarean section, their childbirth history since 2006 was searched to identify parity**
**Previous PTB**	At least one birth occurring prior to a gestational age of 37 completed weeks during previous pregnancy between 2010 and 2014.
**Previous SGA**	At least one SGA betwenn 2013 and 2014
**Previous tobacco consumption**	**Smoking was identified by specific coding at hospital or by the reimbursement of nicotine replacement treatments before or during pregnancy**<br>F17: Mental and behavioral disorders due to use of tobacco<br>Z716: Tobacco abuse counselling<br>Z720: Tobaco use<br>I731: Thromboangiitis obliterans (Buerger)<br>J41: Simple and mucopurulent chronic bronchitis<br>J42: Unspecified chronic bronchitis<br>J43: Emphysema<br>J44: Other chronic obstructive pulmonary disease

	T652: Toxic effect of tobacco and nicotine<br>N07BA01: Nicotine replacement therapy<br>**AND:**<br>C67: Malignant neoplasm of bladder<br>C16: Malignant neoplasm of stomach<br>C64-66,C68: Malignant neoplasms of urinary tract<br>C22: Malignant neoplasm of liver and intrahepatic bile ducts<br>C25: Malignant neoplasm of pancreas<br>**AND:**<br>N07BA03: nicotine replacement treatments<br>R03BB04, R03BB06, R03BB07: treatment of Chronic Obstructive Pulmonary Disease
**Obesity**	E66
**Previous hypertension**	If women have had at least three dispensations of antihypertensive medication at three different dates in the year preceding the pregnancy (or on two dates, if at least one large package (for 3 months) of antihypertensive drugs was dispensed), or if they were hospitalized with a primary diagnosis mentioning a preexisting hypertension (ICD-10 codes: O10, O11) during pregnancy or postpartum (6 weeks postpartum).
