# Supplementary material for: Ability of municipality-level deprivation indices to capture social inequalities in perinatal health in France: A nationwide study using preterm birth and small for gestational age to illustrate their relevance
Source: BMC Public Health. 2022 May 9;22:919. doi: 10.1186/s12889-022-13246-1 (PMC9082984; doi:10.1186/s12889-022-13246-1)
Supplement: Supplementary file 2 — Additional file 2: Appendix 2. SNDS and National perinatal surveys analyses: Multilevel models. Presentation of all covariables of the different multilevel models used in the NPS and SNDS statistical analyses. [file 12889_2022_13246_MOESM2_ESM.pdf]

Appendix 2: Multilevel models

(a) Description of data : Presentation of all covariables of the six multilevel models used in the National perinatal surveys statistical analyses.

**Covariables**	**Preterm birth**						**Small for gestational age**					
	M1	M2	M3	M4	M5	M6	M1	M2	M3	M4	M5	M6
FDep-quintile	x	x	x				x	x	x			
FEDI-quintile				x	x	x				x	x	x
Household monthly income	x			x			x			x		
Mother's educational level		x			x			x			x	
Mother's individual social deprivation index			x			x			x			x
Maternal age	x	x	x	x	x	x	x	x	x	x	x	x
Parity	x	x	x	x	x	x	x	x	x	x	x	x
Previous PTB	x	x	x	x	x	x						
Previous SGA							x	x	x	x	x	x
Other adverse obstetric history	x	x	x	x	x	x	x	x	x	x	x	x
Mother's birth country	x	x	x	x	x	x	x	x	x	x	x	x
Pre-pregnancy body mass index (BMI)	x	x	x	x	x	x	x	x	x	x	x	x
Cannabis use	x	x	x	x	x	x	x	x	x	x	x	x
Smoking at the third trimester	x	x	x	x	x	x	x	x	x	x	x	x

M1, M2, …M6 are the six models presented in the statistical analysis section.

(b) Description of data : Presentation of all covariables of the multilevel models used in the SNDS statistical analyses.

Covariables	Preterm birth												Small for gestational age											
	M1	M2	M3	M4	M5	M6	M7	M8	M9	M10	M11	M12	M1	M2	M3	M4	M5	M6	M7	M8	M9	M10	M11	M12
Proportion of unemployment	x											x	x											x
Proportion of blue-collar workers		x										x		x										x
Median household income			x									x			x									x
Proportion of persons with upper Secondary school education level				x												x								
Proportion of residences with more than 1 person per room					x							x					x							
Proportion of persons with a low level of education (lower secondary education or less)						x						x						x						x
Proportion of single-parent households							x					x							x					x
Proportion of foreign nationals								x												x				
Proportion of unskilled farm workers									x			x									x			x
Proportion of non-home owners										x		x										x		x
Proportion of the household with no access to a car											x												x	
Maternal age	x	x	x	x	x	x	x	x	x	x	x	x	x	x	x	x	x	x	x	x	x	x	x	x
Parity	x	x	x	x	x	x	x	x	x	x	x	x	x	x	x	x	x	x	x	x	x	x	x	x
Pevious PTB	x	x	x	x	x	x	x	x	x	x	x	x												
Previous SGA												x	x	x	x	x	x	x	x	x	x	x	x	x
Previous Hypertension	x	x	x	x	x	x	x	x	x	x	x	x	x	x	x	x	x	x	x	x	x	x	x	x
Previous tobacco consumption	x	x	x	x	x	x	x	x	x	x	x	x	x	x	x	x	x	x	x	x	x	x	x	x
Obesity	x	x	x	x	x	x	x	x	x	x	x	x	x	x	x	x	x	x	x	x	x	x	x	x
Individual maternal deprivation	x	x	x	x	x	x	x	x	x	x	x	x	x	x	x	x	x	x	x	x	x	x	x	x
CMU-C	x	x	x	x	x	x	x	x	x	x	x	x	x	x	x	x	x	x	x	x	x	x	x	x
