# Supplementary material for: Ability of municipality-level deprivation indices to capture social inequalities in perinatal health in France: A nationwide study using preterm birth and small for gestational age to illustrate their relevance
Source: BMC Public Health. 2022 May 9;22:919. doi: 10.1186/s12889-022-13246-1 (PMC9082984; doi:10.1186/s12889-022-13246-1)
Supplement: Supplementary file 3 — Additional file 3: Appendix 3. Maps of the quintiles of the municipalities according to FDep and FEDI indices ; Distribution of municipalities’ urban–rural status according to the quintiles of FDep and FEDI. Classification of municipalities in the various quintiles of FDep and FEDI and Distribution of municipalities’ urban–rural status according to the quintiles of FDep and FEDI. [file 12889_2022_13246_MOESM3_ESM.pdf]

Appendix 3: Maps of the quintiles of the municipalities according to FDep and FEDI indices; Distribution of municipalities' urban–rural status according to the quintiles of FDep and FEDI.

(a) **Description of data :** classification of municipalities in the various quintiles of FDep and FEDI : The FEDI tended to classify urban municipalities in more deprived quintiles, whereas the FDep tended to classify them in less deprived quintiles.

FDep

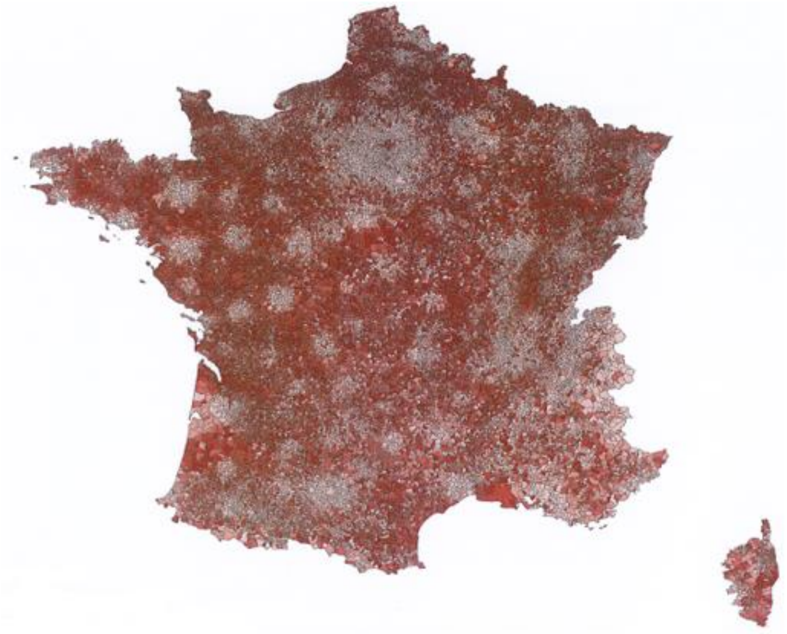

FEDI

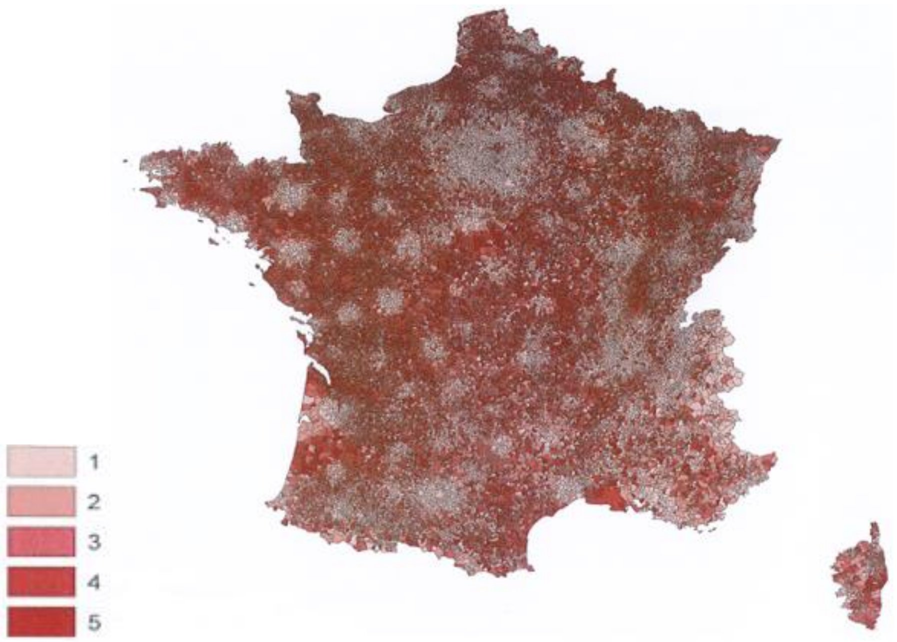

Quintiles of FDep/FEDI

(b) Description of data : Distribution of municipalities' urban–rural status according to the quintiles of FDep and FEDI.

**status of municipalities***		**Q1**		**Q2**		**Q3**		**Q4**		**Q5**	
Isolated rural with very low density	Fdep	6.5	529	16.1	1307	21.3	1728	28.3	2292	27.7	2243
	FEDI	13.5	1091	18.3	1480	22.0	1782	23.9	1936	22.3	1805
Isolated rural with low density	Fdep	2.5	202	12.3	989	20.25	1634	33.0	2665	32.0	2581
	FEDI	8.7	700	16.9	1360	22.4	1810	26.7	2158	25.3	2043
Rural with low relationship with an employment hub	Fdep	8.1	598	22.0	1626	26.0	1920	27.3	2011	16.6	1226
	FEDI	19.9	1470	23.7	1749	21.9	1615	20.1	1485	14.4	1061
Rural with strong relationship with an employment hub	Fdep	23.0	1649	33.3	2387	23.2	1658	15.3	1092	5.2	374
	FEDI	41.2	2949	24.8	1773	16.6	1187	11.1	791	6.4	460
Suburban (intermediate density)	Fdep	27.1	907	23.4	784	17.4	583	16.0	537	16.2	542
	FEDI	21.5	721	16.6	557	14.6	490	15.1	507	32.1	1078
Urban centre (highly urban)	Fdep	37.6	287	21.7	166	13.4	102	11.9	91	15.5	118
	FEDI	4.6	35	7.5	57	12.0	92	13.6	104	62.3	476

*** :** Urban–rural status of the municipalities in which mothers live was identified using a new French definition in 6 groups. An urban unit is a municipality or a group of municipalities that includes a built-up area of at least 2000 inhabitants and in which no building is farther than 200 m away from its nearest neighbour. Urban units are further categorised as: Highly urban (more than 50% of the population lives in cluster with 1 500 inhabitants per km² or in in cluster with 1 500 inhabitants per km²), and Suburban (more than 50% of the population lives in clusters with 300 inhabitants per km² or in clusters with 300 inhabitants per km²). Rural municipalities are categorised in: i) Rural municipalities with strong relationship with an employment hub (belonging to the catchment area of cities with over 50,000 inhabitants; more than 30% of their employed workers work in this employment hub). ii) Rural with low relationship with an employment hub (belonging to the catchment area of cities with over 50,000 inhabitants; less than 30% of their employed workers work in this employment hub). iii) isolated rural with low density (less than 50% of the population lives in cluster of 300 inhabitants per km²); iv) isolated rural with very low density (more than 50% of the population lives in cluster with 300 inhabitants per km²).
