# Supplementary material for: Ability of municipality-level deprivation indices to capture social inequalities in perinatal health in France: A nationwide study using preterm birth and small for gestational age to illustrate their relevance
Source: BMC Public Health. 2022 May 9;22:919. doi: 10.1186/s12889-022-13246-1 (PMC9082984; doi:10.1186/s12889-022-13246-1)
Supplement: Supplementary file 4 — Additional file 4: Appendix 4. Multivariate multilevel analyses for the FDep components in the SNDS. Association between FDep components and preterm birth and small for gestational age. [file 12889_2022_13246_MOESM4_ESM.pdf]

Appendix 4: Multivariate multilevel analyses for the FDep components in the SNDS.

(a) Association between FDep components and preterm birth (Appendix 4a)

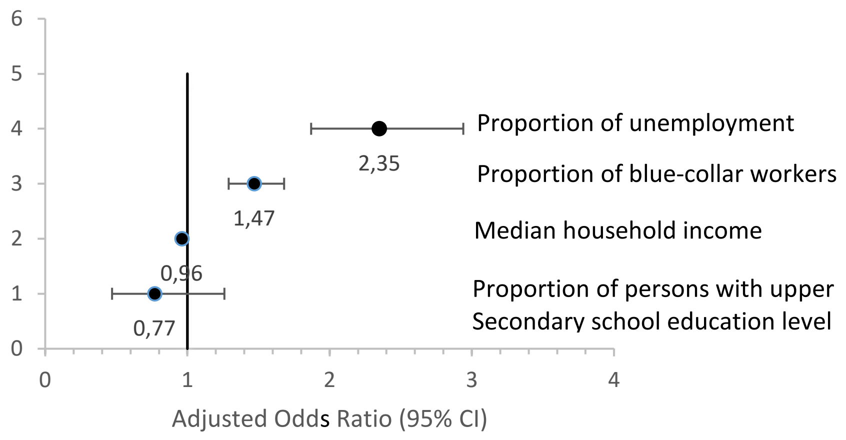

(b) Association between FDep components and Small for gestational age (Appendix 4b)

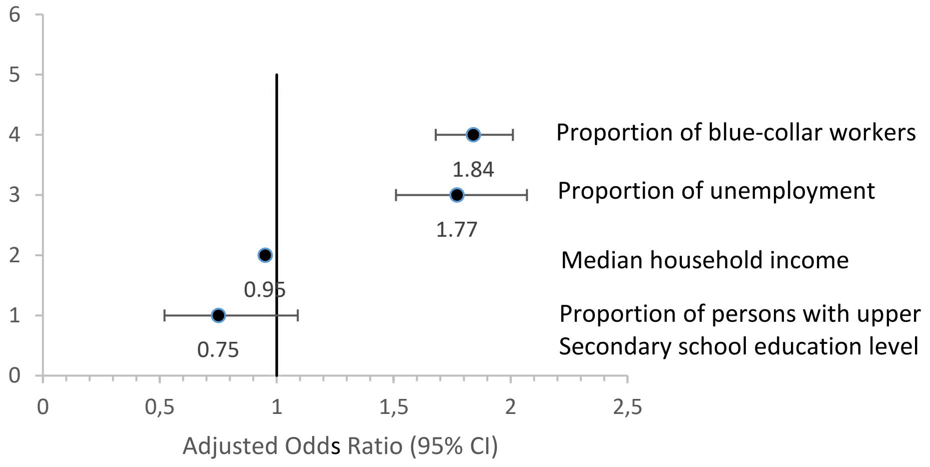

aOR (95% CI) = adjusted odds ratio (95% confidence interval) from multilevel analyses. One component adjusted for individual mother's characteristics per model.
