# Supplementary material for: Ability of municipality-level deprivation indices to capture social inequalities in perinatal health in France: A nationwide study using preterm birth and small for gestational age to illustrate their relevance
Source: BMC Public Health. 2022 May 9;22:919. doi: 10.1186/s12889-022-13246-1 (PMC9082984; doi:10.1186/s12889-022-13246-1)
Supplement: Supplementary file 5 — Additional file 5: Appendix 5. Multivariate multilevel analyses for the FDep components in the SNDS. Association between FEDI components and preterm birth and small for gestational age. [file 12889_2022_13246_MOESM5_ESM.pdf]

Appendix 5: Multivariate multilevel analyses for the FEDI components in the SNDS:

(a) Association between FEDI components and preterm birth (Appendix 5a)

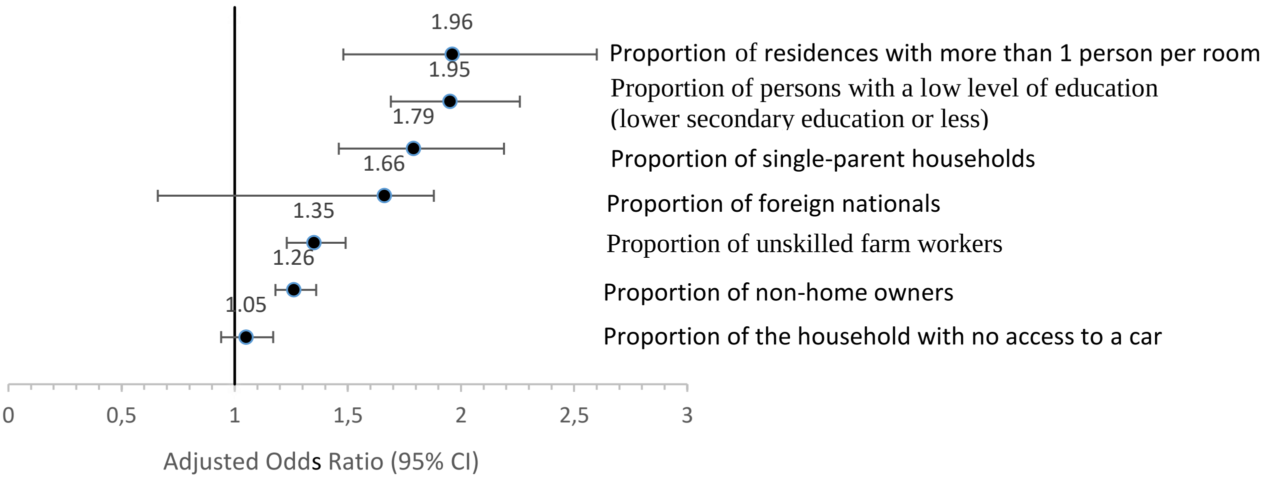

(a) Association between FEDI components and small for gestational age (Appendix 5b)

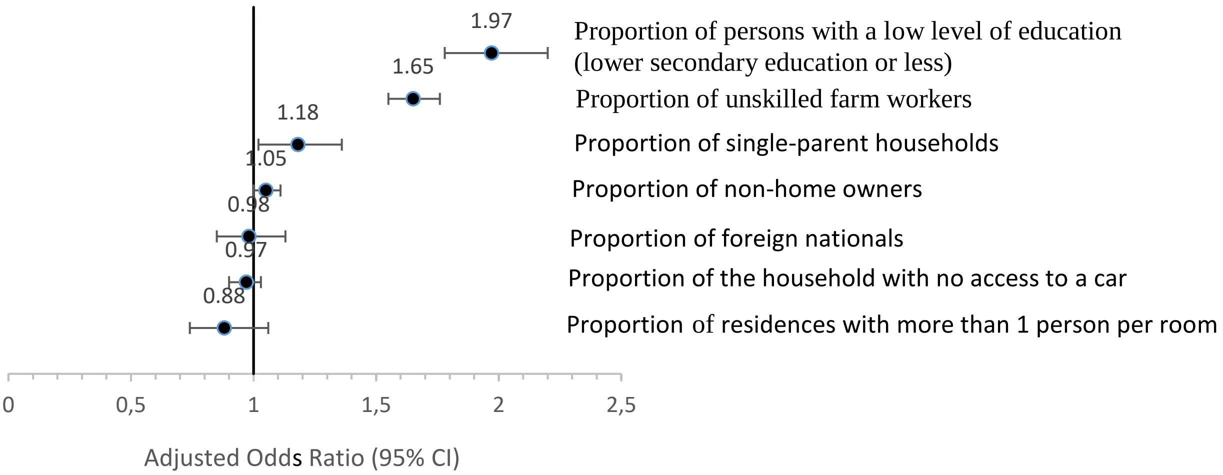

aOR (95% CI) = adjusted odds ratio (95% confidence interval) from multilevel analyses. One component adjusted for individual mother's characteristic per model.
